# Talks with Nurses

**Published:** 1888-02-04

**Authors:** M. M. Basil

**Affiliations:** Surgeon to the Southern Hospital, Manchester


### Talks with Nurses.

By M. M. Basil, M.A., M.B., Surgeon to the Southern Hospital, Manchester.

In the following "Talks" the subject of nursing will be considered in its widest bearings: the hygiene of the sick-room, the routine work of the nurse in medical and surgical cases, the especial nursing of fevers, of children, of mental diseases, will all come under consideration. The "Talks" will consist of expansions of a series of "Notes"—"Notes" which have been used for several years in teaching the subject to a hospital staff of nurses. Throughout the series a sharp line will be maintained between the work of the medical attendant and the duties of the nurse. I must express my indebtedness to nearly a score of writers on the subject. The "Notes" were first drawn up for private teaching, and many books were placed under contribution. I must, however, particularly mention Miss Nightingale's *Notes on Nursing*, to which, in common with many writers, I am especially indebted.

### Ventilation.

It is said that when the Duke of Wellington, after years of warfare and experience in equipping armies, was asked his opinion as to the first requisite for making an efficient army, he answered "boots," the second requisite was "boots," the third one was yet "boots." If we are sure of one point in the physiology of human beings, whether sick or healthy, it is of the necessity of fresh air under all conditions, but especially during illness. A first requisite then of good nursing is fresh air, a second, more fresh air, and a third, yet more fresh air. You must look upon pure air as an essential part of the nourishment of your patient, and take means to supply it as regularly as his food, stimulant, and medicine. Indeed, it is the most essential article of supply which is taken into the human body. We must all, like the chameleon, suck the air, whether poison-crammed or filth-crammed. As nurses it will be your duty to see constantly that your patient is not compelled to inhale any but pure air.

In order to impress on you the importance of this lesson, I shall examine briefly a few facts which would be quite startling, were it not that they are unfortunately so common. Were a beginner in hospital work to be asked the questions, at what season of the year are we most likely to have infectious diseases spreading or breaking out? when are wounds most apt to go wrong; when are patients most likely to get erysipelas, pyæmia, and septicæmia, &c.? he would be inclined to suppose that in the surgical wards, at any rate, the hot season would be the most dangerous one.

The facts are generally the reverse of this natural supposition; most wounds go wrong during cold weather, and it is still more startling to hear that a similar law holds in the tropics. In Calcutta, for instance, the only three pleasant months in the year—viz., November, December, and January—are just the months when the surgical wards are the unhealthiest. When the heat of the summer is all but unbearable we have, under ordinary precautions, but little risk of epidemics on the surgical side. The reason for this state of affairs is as plain as it is painful. During cold weather nurses and patients block up every means of entrance of fresh air into the ward. Through inefficient ventilation it is thus made into a forcing house for poisonous germs. We get the air saturated with sewage, so that it practically becomes fœtid, and the sick are poisoned by their own breath and emanations. You can thus understand that in hospitals where the sick are crowded together great harm may be done to the patients, and many may die simply of the hospital and of the nursing, unless you constantly and conscientiously attend to the thorough ventilation of the ward.

The hospital must not be allowed to become a pest-house. If the ward has no means of securing ventilation and warmth combined, it is safer generally to sacrifice temperature to freshness, and by means of *checked* windows, &c., obtain thorough ventilation independently of the will of the patients or of the nurse.

Other startling facts which impress on us the necessity of paying proper attention to ventilation and other requirements of healthy living are such as these: Prisons and asylums are now among the healthiest places in the kingdom. Not half a century ago they were the centres for originating and spreading infectious diseases. Armies of picked healthy men took consumption at a much higher rate than the average population of the country. Now the condition is nearly equalised, and at no distant period it will, no doubt, be reversed. Want of fresh air, it has been said, is perhaps the cause of half the diseases in the world.

The first rule of nursing, therefore, is, in the words of Miss Nightingale, "to keep the air within as pure as the air without, by night as well as by day, without chilling the patient." Strictly speaking, this is all but an impossibility; and the directions laid down by her, if followed to the letter, would make all rooms uninhabitable. "We cannot demand," says the late Dr. Parkes, "that the air of an inhabited room shall be absolutely as pure as the outside air, for nothing short of breathing in the open air can ensure perfect purity at every respiration. In every dwelling-room there will be some impurity of the air." However, no trace of odour or difference between the room and the outside air in point of freshness should be observed by the nose.

To secure this fresh state of the air several points must be borne in mind. In the first place, a hot room is not necessarily a close room, and conversely a room may be uncomfortably cold and yet anything but fresh. Then, draughts in a room do not mean that it is well ventilated. Vent does not mean ventilation. It is quite possible to give a patient fresh air without chilling him. He is often chilled fatally without getting fresh air. The room can be ventilated properly only by air coming from outside the house. Corridors, stairs, etc., are generally the ventilating shafts that carry out the foul air of the house; hence, open doors are, as a rule, useless as a means of ventilation. As a general principle, windows, more or less modified in structure, must act as the main inlets of fresh air into the sick-room. The window is made to open, the door to shut. They should never be left open together for any length of time. The question as to when the windows should be opened, you will look upon as meaningless. The proper position for a window, or its substitute for admitting air, is to be open; when it should be closed is a more rational query, as that is the exceptional condition. The windows may be opened one, two, three, or four inches from the top. In many rooms arrangements are now in use to prevent draughts. A louvred pane of glass is so simple that it is a pity to find any room without it. For securing the proper degree of freshness of air windows and ventilators should reach well up the ceiling. Unfortunately, an arrangement the reverse of this still holds in many parts of England. Nearly all Lancashire houses have this defect. If there are any especial ventilators, you must see that they are in use; thus, for instance, no books, papers, etc., should cover the opening of a Tobin's tube. The shaft of the chimney should never be obstructed, and, as we shall see, you should always secure a good fire in the sick-room as a means of ventilation. The head of the patient should never be higher than the throat of the chimney. It is also of some importance to remember that the green parts of plants, when exposed to the action of sunlight, are valuable purifiers of air. In the night, however, they give out impurities to the atmosphere, and should therefore never be left in the sick-room after sunset. Flowers have not the same unhealthy property, and are useful in the sick-room, not only on account of their beauty and cheerful appearance, but also because they are genuine destroyers of infection.

In doing house nursing or district nnrsing among the poor, you will often have to exercise much tact and ingenuity to secure proper ventilation. Several simple means may be resorted to, such as the following:

(1) The Candian system, or that of Hinckes Bird. Raise the lower half of the window two to four inches, and place a board under the lower sash. India-rubber cushions may be nailed above and below the board.

(2) Small holes may be drilled between the lower sash of the upper half and the upper sash of the lower half of the window.

(3) You will often find that in the end the kindest thing you can do to your patient is to get a pane of glass smashed "accidentally," and then take all legitimate means to prevent its repair during the illness.

The question of ventilation through the night is important. A good deal of nonsense has been talked about the dangers of "night air." Night air is not dangerous in most parts of England; perhaps it is the only fairly pure air admissible into houses in many of the manufacturing towns of the North. The days when night air in England meant ague with its long train of evils are over, and though their remembrance is still with us, night air can, happily, with proper precautions, be made just as wholesome as day air.

In your endeavour to secure proper ventilation you will often meet with opposition from fear of draughts, which figure very largely in the popular imagination. You will often find draughts complained of persistently, when a lighted candle or a light feather shows absolutely that there is no undue current of air in the room. In most cases such draughts mean proximity to a cold wall. A stone or brick wall reduces the temperature of the body close to it. A large slab of ice would do the same even if the room were nearly air-tight. The draughts sometimes complained of in churches which are almost stifling are of this nature. It is important to remember this fact, as I have known every hole and cranny in a church carefully stuffed under the direction of the doctor, because he was sure he felt draughts while standing next to a cold stone wall. Such a condition must be remedied by removal from the neighbourhood of the wall, by placing a screen between the patient and the wall, or by arrangements like the old arras curtains against the wall.

*(To be continued.)*

